# Dissociative recombination of H_3_^+^: 10 years in retrospect

**DOI:** 10.1098/rsta.2012.0020

**Published:** 2012-11-13

**Authors:** Mats Larsson

**Affiliations:** Department of Physics, AlbaNova University Center, Stockholm University, 10691 Stockholm, Sweden

**Keywords:** recombination, dissociation, Jahn–Teller, electron capture

## Abstract

The dissociative recombination of 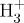 has been an intriguing problem for more than half a century. The early experiments on 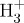 during the first 20 years were carried out without mass analysis in decaying plasma afterglows, and thus the measured rates pertained to an uncontrolled mixture of 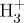 and impurity ions. When mass analysis was used, the rate coefficient was determined to be an uneventful value of about 10^−7^ cm^3^ s^−1^, a very common rate coefficient for many molecular ions. But this was not the end of the story, not even the beginning of the end; it marked only the end of the beginning. The story I will tell in this article started about 10 years ago, when the dissociative recombination of 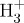 was approaching its deepest crisis. Today, owing to an extensive experimental and theoretical effort, the state of affairs has reached a historically unique level of harmony, although there still remains many things to sort out.

## 1. Introduction

This 10 years retrospective on the dissociative recombination of 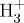 is of course related to the fact that the first Royal Society Discussion Meeting (RSDM) on 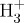 was held in the year 2000, i.e. just over 10 years ago. At that time, which is very clear from a reading of my summary of the situation concerning experimental and, to some extent, theoretical studies of the dissociative recombination of 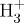 [1], the situation was bewildering. The reaction itself appears, naively, to be very simple:
1.1
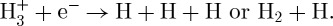

Only three protons and three electrons, of which one electron is free before the reaction and all electrons are bound after the reaction, at the expense of a complete or partial break-up of a stable molecular ion.

At the first RSDM, one can summarize the situation as follows.

— There was consensus that the rate coefficient (*k*_e_) at an electron temperature of 300 K as measured in ion storage rings was about 10^−7^ cm^3^ s^−1^ and that the 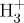 ions used in these experiments occupied only their zeroth vibrational level [1–5]; the preliminary results of Jensen *et al.* [4] and Kreckel *et al.* [5] were reported in [1] as ‘private communications’).
— Theoretical calculations were unable to come anywhere close to the storage ring results [6], typically yielding two orders of magnitude lower rate coefficients. Theory had excluded the direct dissociative recombination mechanism through a favourable curve crossing already through the pioneering works by Kulander & Guest [7] and Michels & Hobbs [8], and the work by Orel *et al.* [6] clearly pointed towards an indirect mechanism dominated by bound Rydberg states. This made 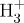 together with HeH^+^ [9,10] atypical ions in terms of dissociative recombination.
— The monitoring of a decaying hydrogen plasma either by measurements of the 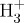 infrared spectrum [11] or by measurement of the electron density by a Langmuir probe in a stationary [12] or flowing afterglow [13–17] gave rate coefficients covering the stunning range from 1.8×10^−7^ to 10^−11^ cm^3^ s^−1^. The early theoretical estimate by Michels & Hobbs [8] no doubt had an influence on the interpretation of the afterglow results, and was sufficiently convincing as to leave even Bates in a state of uncertainty [18].
— The observation of 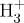 in diffuse interstellar clouds [19], where the ion is destroyed primarily by dissociative recombination, was difficult to reconcile with a rate coefficient of the order of 10^−7^ cm^3^ s^−1^ [20].
— Measurements of isotopologues of 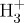 by different techniques also presented a confusing picture.
— Essentially nothing was known about a possible rotational dependence of the rate coefficient.

## 2. The clouds are gathering

If the situation was confusing during the first RSDM, it would get worse. First out were new measurements by means of the stationary afterglow technique [21]. The paper was submitted about six months after the first RSDM on 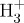 and thus not included even as a personal communication in the review of recombination rate coefficients [1]. It was surprising in several respects. It represented a revival of the stationary afterglow technique, which had not been used since 1984 [12] to study the dissociative recombination of 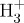. Groups involved in afterglow measurements had, by the turn of the millennium, migrated to the flowing afterglow technique. More surprising, however, was that the measured rate coefficient, less than or equal to 1.3×10^−8^ cm^3^ s^−1^, was about a factor of 10 lower than the one measured by Macdonald *et al.* [12]. Glosik *et al.* [21] pointed out the excellent agreement with the flowing afterglow/Langmuir probe result obtained by Smith & Spanel [15], but, surprisingly, offered no explanation as to why their result differed so much from a result obtained with a very similar stationary afterglow technique [12]. However, it was clear that Glosik *et al.*’s low rate coefficient was difficult to refute.

During the same time, it became clear that the rotational temperature in the ion storage ring experiments needed to be addressed. At CRYRING in Stockholm, several experiments using different ion source conditions in a hollow cathode source were performed during the spring of 2001 and presented at the symposium on dissociative recombination in connection with the American Chemical Society meeting in Chicago in August 2001 [22]. The rate coefficient depended on the ion source conditions (pressure, gas mixture), and hence, probably, on the rotational distribution, but the experiment was not well characterized and many questions remained, one of them obviously being: is it possible that the rate coefficient decreases radically when the rotational temperature is decreased?

**Figure 1.**
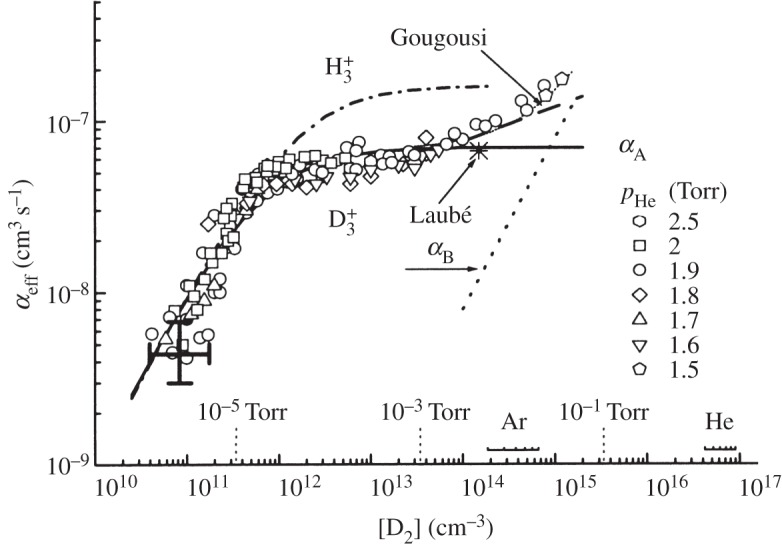
Recombination of 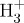 and the dependence of the observed effective rate coefficient (*α*_eff_) on the H_2_ density. The measurements in the stationary afterglow apparatus were performed at various helium pressures in the range of 1.5–3 Torr and at several Ar densities (indicated on the *x*-axis). The He temperature was 260±40 K. The crosses indicate error bars. Results from Laubé *et al* [17], Gougousi *et al.* [16], Amano [11] and Canosa *et al.* [14] are included. Adapted from Plasil *et al.* [23].

The Chicago meeting also included a presentation by Glosik and co-workers [23], in which the recombination rate coefficient for 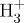 was lowered to less than 3×10^−9^ cm^3^ s^−1^. Figure 1 shows the recombination rate coefficient as a function of the D_2_ density (for some reason, it was the D_2_ density curve which was shown; however, an essentially identical curve was recorded for H_2_) in the stationary afterglow experiment taken from Plasil *et al.* [23]. This figure was to play the key role during the coming years in Glosik’s arguing for a very low, maybe negligible, recombination rate coefficient for 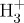
and 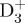. The argument was that H_2_ (and D_2_) contributed to the deionization process at densities above 10^11^ cm^−3^ by three-body collisions by stabilizing the electron-capture process:
2.1
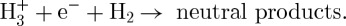

Not only did this explain, according to Glosik, the very low binary (in the absence of H_2_) recombination rate coefficient, it also explained why other afterglow experiments gave higher values; these experiments [16,17] had been carried out at H_2_ densities where the three-body effect had saturated. To the best of my recollection, none of the afterglow researchers present at Glosik’s presentation publically objected to his interpretation.

The Chicago meeting also witnessed a presentation of a new theoretical calculation by Greene *et al.* [24], which included a new mechanism based on the Jahn–Teller coupling [25]. Their preliminary results were a factor of 10 smaller than the storage ring results and in agreement with Glosik’s afterglow results [21]. Kokoouline *et al.* [26] published their results in *Nature*, and the letter to *Nature* was followed by a ‘New and Views’ by Suzor-Weiner & Schneider [27] who remarked (concerning rotational excitations) that: ‘Moreover, the effect should increase for rotationally hot 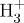 target ions, perhaps explaining the larger value measured ion storage ring experiments’ (p. 872).

McCall & Oka [28], p. 370 pointed out that a low recombination rate was straight-forward to reconcile with the observation of 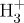 in diffuse interstellar clouds:

perhaps *k*_e_ [assuming a rate coefficient of about 10^−7^ cm^3^ s^−1^] is wrong, in which case the cosmic-ray flux and electron fraction in diffuse clouds are what we expect them to be. But perhaps the current value of *k*_e_ is correct! Then there is exciting new astrophysics waiting to be explored… either carbon is mostly not ionized in diffuse clouds or the cosmic-ray flux is much higher than generally thought.

Given the confusing situation in 2001 regarding the rate of recombination of 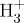 and its important astrophysical implications, Oka’s cry for help [29] is understandable! But the clouds would soon disperse.

## 3. The clouds are dispersing

The 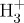 community responded vigorously to the challenge, and the next 5 years saw the most intense activity, experimental and theoretical, in trying to solve the riddle with the dissociative recombination of 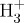. At the second RSDM on 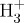, Oka cautiously noted that it seemed like the results from ion storage ring experiments and theoretical calculations were converging [30].

In the proceedings of the Chicago meeting, one can note some optimism in the contribution by Larsson *et al.* [22], who are even (concerning preliminary results from June 2001) talking about ‘… a breakthrough in the experimental study of the DR [dissociative recombination] 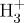 …’ (p. 90). In fact, the CRYRING team was on the wrong track and it was not until it joined forces with the Saykally group at UC Berkeley that the breakthrough would emerge [31–33]. A supersonic expansion ion source was built and characterized at Berkeley, shipped to Stockholm and CRYRING for preliminary tests, shipped back to Berkeley for modifications and finally shipped back to Stockholm for experiments. The characterization of the rotational temperature of the 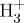 ions extracted from the ion source by means of cavity-ring-down spectroscopy showed that the ions were rotationally cold. The experiments at CRYRING gave a much more structured cross section than was obtained in earlier experiments, thus showing that the ions were colder than in any previous experiment [31–33].

**Figure 2.**
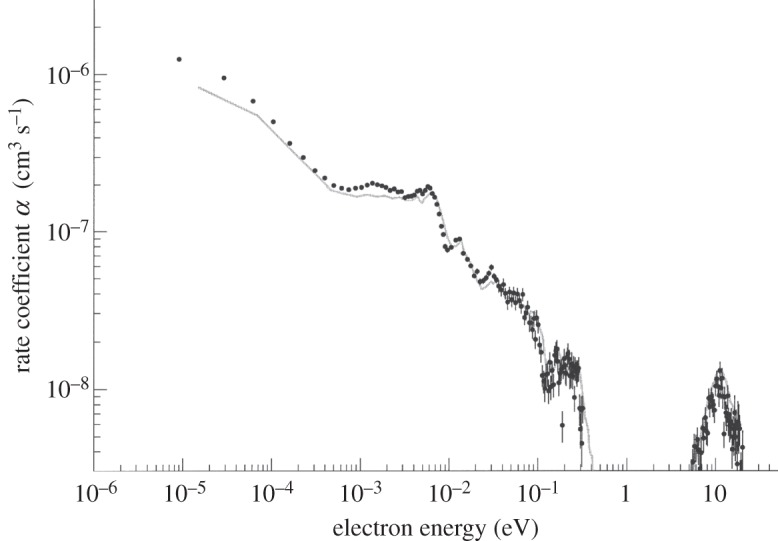
Comparison of results for dissociative recombination of 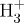. The black dots displays the preliminary results from TSR [35], and the grey line displays the results from CRYRING [31–[33]. The results on the *y*-axis are the rate coefficient measured in the merged electron and ion beams. The small difference between the experimental data of CRYRING and TSR is due to the colder electron beam used in the TSR experiment (see [36] for a detailed discussion of the resolution merged-beam experiments). (H. Kreckel 2004, personal communication).

In any type of experimental physics endeavour, it is highly desirable that experimental results from one experiment are reproduced by another, completely independent, experiment. The efforts at TSR in Heidelberg, which had been proceeding in parallel with those at CRYRING for quite some time, were to play a decisive role. Kreckel *et al.* [5], in their experiments measuring the 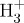 vibrational distribution as a function of storage time in TSR, had noted that their data were best fitted when a substantial rotational energy, 0.3 eV, in the 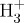 ions was assumed. Lammich *et al.* [34] carried out extensive experiments on the isotopologue D_2_H^+^ and found that the cross section depended on the degree of rotational excitation. The TSR team now pushed for an elegant solution to the problem of rotational excitations. They used a 22-pole radio-frequency ion trap cooled to 10 K as injector to the storage ring, and used the ultracold (0.5 meV transverse energy spread) as a target in the recombination experiments. Instead of reproducing the final results published by Kreckel *et al.* [35], the preliminary results, distributed to the 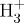 community in December 2004, are shown in figure 2 because it more immediately conveys the excitement we felt when we realized that the results from the two storage rings were in perfect agreement. It should be noted that the TSR result was not an absolute measurement but a relative one, normalized to the CRYRING result for high-energy peak at 10 eV. The small differences below 10 meV are entirely due to the small difference in electron temperature (see [36] for a detailed analysis of the electron temperature influence on the resolution in storage ring experiments). Recent experiments at the TSR gave absolute values in excellent agreement with the CRYRING results [37].

**Figure 3.**
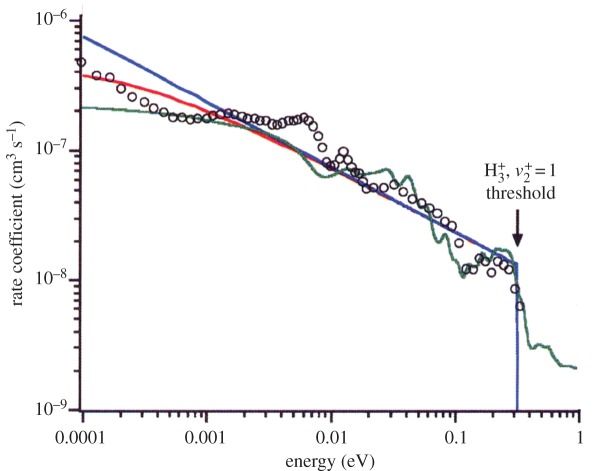
The rate coefficient (in a merged electron–ion beam with parameters from the TSR electron target) for dissociative recombination of 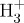. Circles: experimental results from Kreckel *et al.* [35]; blue solid line: unconvolved theoretical results from Jungen & Pratt [40]; red solid line: theoretical results from Jungen & Pratt [40] convolved with the parameters for the electron target in TSR [35]; green solid line: convolved theoretical results from Fonseca dos Santos *et al.* [41]. Adapted from Jungen & Pratt [40].

Already when the CRYRING experiments with the supersonic expansion source were ongoing, the CRYRING team had been informed by Greene that he and his collaborators had found a convention inconsistency in their calculations [26], which, when corrected, increased the cross section by a factor of *π*^2^, i.e. essentially a factor of ten. This brought the theoretical cross section in overall agreement with the storage ring results [38,39]. Later, Jungen & Pratt [40] would use a combined analytical and empirical approach to arrive at essentially the same result. Figure 3 illustrates the excellent agreement between experiment and theory.

Meanwhile Glosik’s group launched a series of experiments using the stationary afterglow technique [42–44], and claimed that the negligible recombination rate coefficient (at low H_2_ density; figure 1) was in agreement with theory. This situation changed in 2003, with two publications [38,39]. The Glosik group also performed experiments using a flowing afterglow apparatus [45,46] and cavity-ring down absorption experiments [47–49]. The Glosik group slowly realized that upholding a very low binary recombination rate coefficient was difficult to reconcile with the very good agreement between theory and ion storage ring experiments. Johnsen [50] offered a speculative but plausible explanation of the peculiar H_2_ density dependence shown in figure 1; if 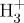 (*v*=1) recombined much slower than 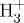 (*v*=0), incomplete vibrational quenching of the 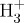 ions due to insufficient H_2_ density would leave many slowly recombining 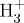 (*v*=1) ions in the plasma. The advantage with this hypothesis was that it could be tested theoretically, and this was done a few years later by Fonseca dos Santos *et al.* [41], who effectively falsified Johnsen’s hypothesis; 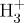 (*v*=1) recombines even faster than 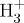 (*v*=0).

The next wave of publications from the Glosik group [51–56] focused on understanding the region above a D_2_ (H_2_) density of about 10^12^ cm^−3^ and concluded that the binary recombination rate coefficient was essentially in agreement with theory and the storage ring results. They were unable to explain the decrease in the rate coefficient below 10^12^ cm^−3^.

At the 8th International Conference on Dissociative Recombination: Theory, Experiments and Applications in Lake Tahoe in 2010, Johnsen [57] discussed the 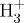 plasma experiments in the Glosik group, and in their recent review of dissociative recombination of 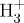, Johnsen & Guberman [58] proposed an explanation that appears both simple and reasonable. At 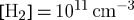, they estimated that the time scale for production of 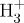 ions in the stationary afterglow experiment was 10 ms, whereas the time scale for destruction was 1 ms. Thus, the loss rate of electrons, which is the quantity measured in an afterglow experiment, was limited not by recombination but by the formation rate of the ions. I steer the reader to Johnsen & Guberman [58] for a critical analysis of all afterglow experiments on 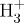. Their conclusion was that there are no afterglow experiments supporting a recombination rate coefficient for 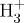 (*v*=0) significantly smaller that obtained in the storage rings.

## 4. The isotopologues and nuclear spin effects

Kokoouline and co-workers [39,59] and Greene & Kokoouline [60] studied the isotopologues 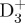, D_2_H^+^ and H_2_D^+^ and found good agreement with the CRYRING results [61] for 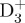, fairly good agreement with the CRYRING results for H_2_D^+^ [62], and quite poor agreement with the TSR results for D_2_H^+^ [34]. The question that arose was whether the disagreement for D_2_H^+^ depended on a flawed experiment or problems with the theory. In order to test this, we performed experiments at CRYRING and obtained perfect agreement with the results from the TSR [63]. Pagani *et al.* [64] included new calculations by Kokoouline, which included more rovibrational states and also improved numerical treatment of symmetrization of rovibronic wave functions of D_2_H^+^, something that brought the results into better agreement with the experimental results.

Kokoouline & Greene [39] initially found that ground state 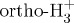 recombines faster than 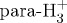; however, in more refined calculation, Fonseca dos Santos *et al.* [41] found a distinctly larger recombination cross section for 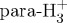. Experiments at TSR [35] seemed to support this, although the difference in rate between para and ortho was much smaller, and subsequent experiments at TSR were non-conclusive [65]. Experiments at CRYRING in which highly enriched 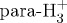 was used showed that 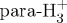 clearly recombined faster than 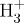 produced from normal H_2_ [66]. The ratio of the rate coefficients of pure 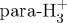 to pure 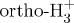 was determined to be approximately 2 at low collision energies, which is too small a ratio to make dissociative recombination the dominant process in determining the ortho/para ratio of 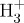 in the diffuse interstellar medium [66]. A more careful analysis has since then modified this statement, suggesting that the ortho/para 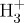 ratio is controlled by a competition between dissociative recombination and thermalization via reactive collisions with H_2_ [67].

The para/ortho ratio has also been studied in the plasma afterglow [68,69], where a para/ortho rate coefficient ratio of approximately 10 or more if the error bars are taken into account was found at an electron temperature of 77 K, and Kokoouline and Greene’s theoretical ratio at 77 K was 2.6 [64].

## 5. Conclusions

I have attempted to describe some of the key advances in the study of the dissociative recombination of 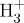 since the first RSDM on 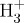 12 years ago. Space limitations have made it impossible to be comprehensive. A book chapter in the research monograph by Larsson & Orel [36], a feature article [70], and the recent review by Johnsen & Guberman [58] fill the gaps in this article, and the latter is in particular noteworthy for its critical analysis of the plasma afterglow experiments and the theory of dissociative recombination of 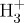.

Major experimental and theoretical efforts during the past decade have brought the situation with 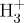 dissociative recombination to a reasonable degree of satisfaction. Although 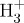 has been famous (or maybe infamous) for delivering surprises, it seems unlikely that there will be any major changes in the 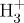 recombination framework. This does not, however, mean that there are no remaining question marks to iron out. Remaining questions are:

— the analysis of the plasma afterglow experiments has not yet been exhausted; and
— the finer details in the comparison of storage ring results and theory is not yet satisfactory. The resonances observed in the experiments at storage rings (figure 2) have a smaller amplitude than those calculated by theory. A possible explanation for this was put forward by Petrignani *et al.* [71], who, in a very careful and systematic study using different types of ion sources at the TSR, found that the rotational temperature experiments with storage rings claimed to have been carried out with cold ions (approx. 50 K) probably were done with ions having a rotational temperature around 300–400 K owing to poorly controlled heating mechanisms. The recombination rate coefficient’s dependence on the rotational quantum number is sufficiently small to render the effect to be of only minor astrophysical importance; however, it suggests the desirability to develop an *in situ* measurement of the ion temperature in a storage ring.

To me, there is nevertheless something a little sad about ending an article by concluding that the long and controversial story of the dissociative recombination of 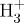 is approaching its final chapter. It gives me the same slightly sad emotion as each time I have been watching Mozart’s *Cosi fan tutte*, his last great comic opera, and the curtain falls. Just as the belief that Mozart could have written another *Cosi fan tutte* if he had lived is a saddening thought, it is also a saddening thought to believe that 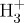 cannot deliver yet another major surprise to its many friends. But maybe it can!
